# Transport of Ciliary Membrane Proteins

**DOI:** 10.3389/fcell.2019.00381

**Published:** 2020-01-13

**Authors:** Huan Long, Kaiyao Huang

**Affiliations:** Key Laboratory of Algal Biology, Institute of Hydrobiology, Chinese Academy of Sciences, Wuhan, China

**Keywords:** cilia, ciliary membrane protein, diffusion barrier, ciliary ectosome, ciliopathies

## Abstract

Cilia and flagella are highly conserved organelles in eukaryotic cells that drive cell movement and act as cell antennae that receive and transmit signals. In addition to receiving and transducing external signals that activate signal cascades, cilia also secrete ciliary ectosomes that send signals to recipient cells, and thereby mediate cell–cell communication. Abnormal ciliary function leads to various ciliopathies, and the precise transport and localization of ciliary membrane proteins are essential for cilium function. This review summarizes current knowledge about the transport processes of ciliary membrane proteins after their synthesis at the endoplasmic reticulum: modification and sorting in the Golgi apparatus, transport through vesicles to the ciliary base, entrance into cilia through the diffusion barrier, and turnover by ectosome secretion. The molecular mechanisms and regulation involved in each step are also discussed. Transport of ciliary membrane proteins is a complex, precise cellular process coordinated among multiple organelles. By systematically analyzing the existing research, we identify topics that should be further investigated to promote progress in this field of research.

## Introduction

Cilia and flagella are both organelles that protrude from the surface of eukaryotic cells. Because of the structural and functional similarities between cilia and flagella, the two terms are often used interchangeably. Cilia are composed of the ciliary membrane, central axoneme, and matrix, and are classified into two main types, motile cilia and primary cilia, based on the organization of their axonemes. The axonemes of motile (9 + 2) cilia contain nine outer doublet microtubules, a central pair of singlet microtubules, dynein arms, radial spokes, and other protein complexes required for motility, whereas primary cilia lack most of these structures and typically have axonemes containing 9 + 0 microtubules (Satir and Christensen, 2007; Pedersen and Rosenbaum, 2008).

Cilia are evolutionarily conserved: they are present in single-cell protozoa, and are also widely distributed in vertebrates, including human cells (Rosenbaum and Witman, 2002). The functions of cilia can be summarized into three categories. The first is motility; for example, *Chlamydomonas reinhardtii* cells and mammalian sperm both use flagella as a propeller of movement, and the cilia of human respiratory epithelial cells remove bacteria and dust through directional movement. The second is sensory function; for instance, specialized cilia are essential to vision, hearing, and olfaction in vertebrates (Sobkowicz et al., 1995; Axelrod, 2008). Furthermore, numerous membrane receptors, including those involved in signal pathways such as the Hedgehog and Wnt pathways, which regulate animal development, are concentrated on cilia (Goetz and Anderson, 2010; Oh and Katsanis, 2013). Therefore, like antennae, cilia can receive signals from the outside environment. The third is secretory function (Wood et al., 2013; Wang et al., 2014); ciliary ectosomes are secreted from the ciliary membrane by budding and carry specific protein components, including special ciliary membrane proteins, enzymes, and various signal molecules that ensure communication between cells (Wang et al., 2014; Long et al., 2016). Therefore, Cilia have been proposed to act both as signal receivers and as transmitters (Wood et al., 2013; Wang et al., 2014; Long et al., 2016).

Abnormal ciliary structure and function result in a variety of hereditary diseases, collectively called ciliopathies, that include Autosomal dominant Polycystic Kidney Disease (ADPKD), Retinitis pigmentosa, Nephronophthisis, Bardet-Biedl syndrome, and Joubert syndrome (Adams et al., 2007; Fliegauf et al., 2007; Hildebrandt et al., 2011; Madhivanan and Aguilar, 2014; Braun and Hildebrandt, 2016). Many ciliopathies are caused by the incorrect transport of ciliary membrane proteins, as the sensory function of cilia depends heavily on the many membrane receptors located on cilia. Therefore, the study of the transport and turnover of ciliary membrane proteins is of theoretical and clinical interest, and could provide clues toward the diagnosis and treatment of ciliopathies.

Unlike other organelles such as chloroplasts and mitochondria, cilia are not completely bound by a membrane that separates them from the plasma membrane (PM) and cytoplasm. However, they do possess a specific protein and lipid composition, and proteomic analysis shows that cilia contain at least 600 different proteins (Pazour et al., 2005; Ishikawa et al., 2012). However, there is no evidence of protein synthesis occurring within cilia. Thus, how ciliary proteins are selectively transported into cilia has long been a topic of active research.

The maintenance of ciliary protein homeostasis depends mainly on two mechanisms. First, depending on their molecular weight, some soluble proteins can pass through the “molecular sieve” at the ciliary base, in a process that may be regulated by components of the nuclear pore complex. Proteins smaller than 40 kDa can diffuse freely through the “molecular sieve”, whereas larger cargo proteins need the assistance of nuclear transporters such as the Karyopherins. Karyopherins may interact with cargo proteins by identifying their ciliary targeting sequences. For instance, Importin-β1 and Transportin 1 (TNPO1) from the β-karyopherin family are involved in the transport of the soluble protein KIF17 into cilia (Kee et al., 2012; Obado and Rout, 2012). The direction of transport is regulated by a gradient of Ran GTPase. Second, the diffusion barrier which locates at the transition zone (TZ) membrane regulates the entry and exit of ciliary membrane proteins (Jensen and Leroux, 2017). The TZ comprises an area at the ciliary base about 135 nm long, adjacent to the cell body, which contains an elaborate stellate pattern in the interior of the microtubule barrel (Geimer and Melkonian, 2004; O’Toole et al., 2007; Dutcher and O’Toole, 2016). These stellate patterns are visible as an osmophilic H-shaped structure in longitudinal transmission electron microscopy sections. The TZ also contains Y-shaped linkers positioned between the microtubule core and the ciliary membrane (Dutcher and O’Toole, 2016). The TZ membrane contains two sets of intramembranous particles that form structures known as the ciliary necklace and ciliary bracelet (Weiss et al., 1977; Dutcher and O’Toole, 2016), whose compositions are unknown. The ciliary necklace, whose structure is conserved in different ciliated species, is the location of the diffusion barrier separating the PM from the ciliary membrane (Dutcher and O’Toole, 2016). The process whereby ciliary membrane proteins pass through the diffusion barrier is regulated by the proteins of the BBSome complex (associated with Bardet-Biedl syndrome), the MKS complex (associated with Meckel-Gruber syndrome), the NPHP complex (associated with Nephronophthisis), and Septin 2 (Hu et al., 2010; Cevik et al., 2013; Jensen et al., 2015; Lambacher et al., 2016).

The transport mode for most of ciliary membrane proteins, after their synthesis in the endoplasmic reticulum (ER), involves processing, modification, and sorting by the Golgi apparatus. Next, they are carried to the PM or ciliary base membrane by transport vesicles, and enter the cilia by lateral transport through the diffusion barrier with the help of assistant proteins (such as Intraflagellar Transport B subunits, KIF17, and Rab23 in the lateral transport of D1-type dopaminergic receptors) (Leaf and Von Zastrow, 2015) or through recycling pathway (Boehlke et al., 2010). After proteins enter cilia, their dynamics and homeostasis are maintained by two methods: they enter and exit the cilium through a process known as intraflagellar transport (IFT), or they are secreted to the extracellular environment by ciliary ectosomes. The transport of ciliary membrane proteins is an elaborate, precise process involving the coordination of multiple organelles. Several transport modes corresponding to different types of ciliary proteins had been identified (Hunnicutt et al., 1990; Milenkovic et al., 2009; Boehlke et al., 2010; Kim et al., 2014; Leaf and Von Zastrow, 2015; Witzgall, 2018). In this review, we summarize the current understanding of the process of ciliary membrane protein transport and related regulatory mechanisms, and highlight research hotspots currently in need of further investigation.

## Modification and Sorting of Ciliary Membrane Proteins in the Golgi Apparatus

**FIGURE 1:**
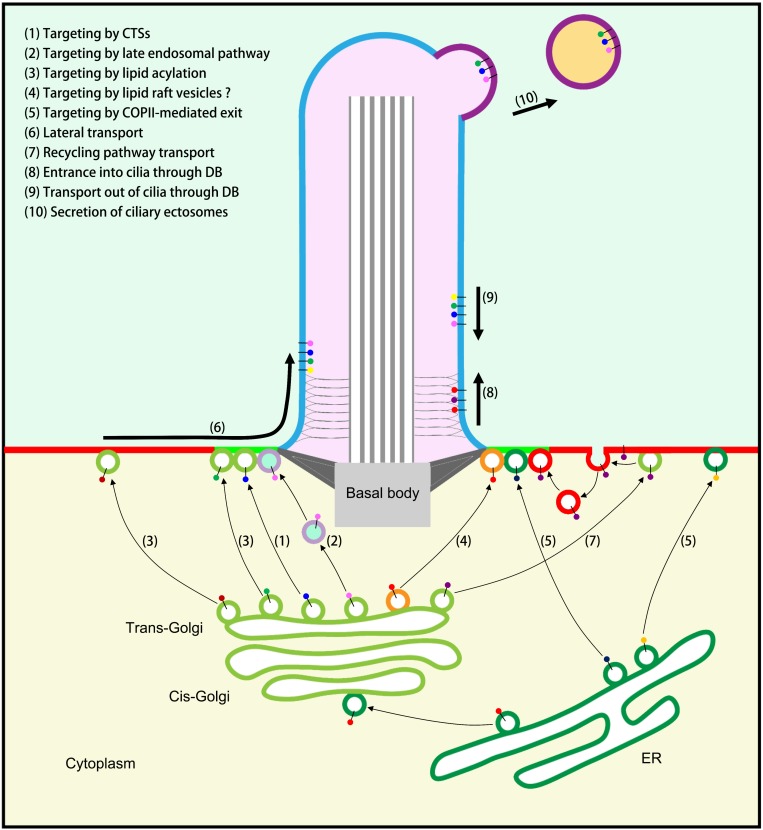
Model of ciliary membrane protein transport. (1) Targeting to cilia by ciliary targeting sequences (CTSs). (2) Targeting to cilia by the late endosomal (LE) pathway. (3) Targeting to cilia or PM by lipid acylation and special carriers. (4) Targeting to cilia by specific lipid raft vesicles (unproven). (5) Targeting to cilia or PM by COPII-mediated exit from the ER. (6) Transport to the PM followed by lateral transport into cilia when cell signaling is activated. (7) Recycling pathway of ciliary membrane proteins that first reach the PM: endocytosis followed by polarized exocytosis at the periciliary membrane. (8) Transport through the diffusion barrier (DB) and entrance into cilia through the IFT-A/TULP3 complex. (9) Transport out of cilia by the IFT-B/BBSome complex. (10) The secretion of ciliary ectosomes regulates ciliary membrane protein homeostasis.

The Golgi apparatus is the intracellular logistics center that receives newly synthesized proteins from the ER. Ciliary membrane proteins are processed, modified, sorted, and packaged in the Golgi and then transported to their various destinations. Many post-translational processes, including glycosylation, lipid acylation, and prenylation, occur at the Golgi: for example, glycosylation of Polycystin 1 and Polycystin 2 (Cai et al., 2014; Kim et al., 2014), myristoylation of NPHP3 and Cystin (Wright et al., 2011), and prenylation of INPP5E (inositol 1,4,5-trisphosphate 5-phosphatase) (Humbert et al., 2012). Then ciliary membrane proteins are sorted and packaged, based on their different properties, into different transport vesicles in the following three ways (Figure 1).

### i. Transmembrane Proteins With Ciliary Targeting Sequences

Numerous types of ciliary targeting sequence (CTSs) have been reported, but they do not share sequence similarity. For instance, CTSs in PKD2, Rhodopsin and CNGB1 (cyclic nucleotide-gated b1) are characterized as Vxp motifs (Deretic et al., 1998; Geng et al., 2006; Jenkins et al., 2006; Mazelova et al., 2009a; Wang et al., 2012); G-protein-coupled receptors (GPCRs) contain (V/I)KARK and Ax(S/A)xQ motifs (Berbari et al., 2008a, b; Badgandi et al., 2017); and INPP5E contains a FDRELYL motif at amino acids 609–615 (Humbert et al., 2012).

The CTSs of ciliary membrane proteins are recognized by specific membrane protein carriers, which then transport the proteins to the cilia. Like Fibrocystin, photoreceptor retinol dehydrogenase, Rhodopsin, and Retinitis pigmentosa 2 interact with TNPO1 through their CTSs to form the TNPO1-Rab8-CTS complex, which mediates the further selective entry of these membrane proteins into cilia and their retention therein (Madugula and Lu, 2016). The tubby-family proteins Tulp3 and tubby (Tub) are carriers in the trafficking of multiple GPCRs into the ciliary membrane (Mukhopadhyay et al., 2010; Sun et al., 2012). The exploration of further carriers or adaptors in ciliary membrane protein trafficking is an important direction for future research.

### ii. The Late Endosomal Pathway Regulates the Ciliary Targeting of Transmembrane Proteins Without CTSs

Newly synthesized Peripherin 2 (PRPH2), a tetraspanin protein concentrated in the light-sensing cilium of vertebrate photoreceptor, is first targeted to the luminal membrane of the late endosomal (LE), and then targeted to cilia through ubiquitination and binding to Hrs, a component of the ESCRT complex (Otsu et al., 2019).

### iii. Lipid-Acylated Ciliary Membrane Proteins Are Transported by Specific Carriers

For example, Unc-119 is responsible for transporting myristoylated NPHP3 and Cystin, and ARL3-GTP releases myristoylated cargo from UNC119 (Wright et al., 2011). A Golgi-localized palmitoyltransferase, DHHC-21, is essential to the palmitoylation-dependent transport of N-terminal dual-acylation proteins to cilia (Kumeta et al., 2018).

There may also be other sorting mechanisms beside these three. Notably, the lipid compositions of the ciliary membrane and the PM are distinct: the phosphatidylinositol in the PM is mainly PIP2, whereas the main phospholipid component of the ciliary membrane is PI(4)P (Jensen and Leroux, 2017). With the help of the MKS complex and the phosphodiesterase 6 delta subunit (PDE6δ), INPP5E is targeted to cilia and in turn recruits PI(3,4,5)P3 and PI(4,5)P2 to the TZ, where they are hydrolyzed to PI(4)P (Fansa et al., 2016; Goncalves and Pelletier, 2017). Studies in *Trypanosoma* have also shown that ciliary membranes have specific lipid compositions, including steroids, glycolipids, and sphingolipids, the typical components of lipid rafts (Maric et al., 2010). Hence, packaging with different lipid raft vesicles might constitute another kind of sorting signal for ciliary membrane protein transport. However, there have been only a few studies in this area, which should be expanded upon in future.

The transport of ciliary membrane proteins from the Golgi to cilia is regulated by a series of proteins, including an IFT complex B subunit, IFT20, that is localized to both the cilia and the Golgi apparatus. Knockdown of IFT20 expression reduces the level of PKD2 in primary cilia (Follit et al., 2006), and IFT20 interacts with the Golgi microtubule-binding protein GMAP210 (Golgi microtubule-associated protein 210) to regulate PKD2 transport. Cells with a deletion of GMAP210 exhibit a ciliary deficiency phenotype and reduced expression of PKD2 (Jonassen et al., 2008). Interaction of Polycystins 1 and 2 (PC1 and PC2) and GPCR proteolytic site cleavage of PC1 are both required for the polycystin complex to reach the *trans*-Golgi network (TGN) and for subsequent ciliary targeting. During this process, a protein complex composed of Rabep1, GGA1, and Arl3 is responsible for sorting and targeting the polycystin complex to the cilium (Kim et al., 2014). The transport of ciliary membrane proteins from Golgi to cilia is also regulated by a series of small GTPase (Rab 5/8/10/11/17/23/28/29/34 and Rabl2/4/5) (Moritz et al., 2001; Wang et al., 2012; Lu and Madugula, 2018). For example, mammalian Rab11-Rabin8-Rab8 cascades are involved in Rhodopsin transport to the photoreceptor cell cilium (Knodler et al., 2010; Westlake et al., 2011). Trafficking of PC1 from the TGN to cilia via post-Golgi vesicles is dependent on the protein Arf4 (Ward et al., 2011). Arl13b is responsible for dissociating PDE6D from the INPP5E-PDE6D complex and further regulating the transport of INPP5E into cilia (Humbert et al., 2012).

Nonetheless, not all ciliary membrane proteins need to be transported through the Golgi apparatus. Peripherin/rds, for example, appears to reach the cilia by a transport route that bypasses the Golgi (Witzgall, 2018). Treatment with the Golgi-disrupting agent brefeldin A, which leads to the collapse of the Golgi apparatus, only marginally affects the trafficking of peripherin/rds to primary cilia (Tian et al., 2014). In addition, treatment with 30N12 or monensin, drugs that inhibit transport from the *trans*-Golgi compartment to the PM, have no effect on the localization of peripherin/rds in primary cilia (Tian et al., 2014). This unconventional ciliary targeting of peripherin/rds is dependent on COPII-mediated exit from the ER (Figure 1) but independent of GRASP55-mediated secretion (Tian et al., 2014). Thus, the Golgi is not the only starting point for the transport of ciliary membrane proteins to cilia.

## Ciliary Membrane Proteins Are Transported Through Vesicles to Reach the Pm or Periciliary Membrane

Vesicles carrying ciliary membrane proteins secreted from the Golgi apparatus are further transported under the action of motor proteins that are members of the dynein and kinesin families. In photoreceptor cells, cytoplasmic dynein mediates the transport of Rhodopsin from the Golgi to the ciliary base (Tai et al., 1999). In non-photoreceptor cells such as retinal pigment epithelial (RPE) cells, KIFC1 (kinesin family member C1) accumulates in the Golgi apparatus after serum starvation. Knockdown of KIFC1 expression inhibits the export of ciliary receptors from the Golgi complex. Overexpression of KIFC1 affects the Golgi localization of GMAP210 and IFT20, which (as discussed above) are involved in membrane protein transport to cilia (Lee et al., 2018). Furthermore, KIFC1 physically interacts with ASAP1 (ADP-ribosylation factor GTPase-activating protein with SH3 domain, ankyrin repeat and PH domain 1), which regulates the budding of rhodopsin transport carriers from the Golgi complex, and KIFC1 depletion causes accumulation of ASAP1 in Golgi (Lee et al., 2018). In this way, KIFC1, a member of kinesin family, regulates the transport of ciliary membrane proteins from Golgi to cilia.

Vesicles loaded with ciliary membrane proteins have two destinations (Figure 1). The first is the PM. Some ciliary membrane proteins – including Agglutinin (Hunnicutt et al., 1990), Smoothened (Smo) (Milenkovic et al., 2009), and the D1-type dopaminergic receptor (Leaf and Von Zastrow, 2015) – are first transported by vesicles to the PM, where they are stored before undergoing lateral transport into cilia when cell signaling is activated. For instance, the binding of the ligand Sonic Hedgehog (Shh) to its receptor Patched 1 (Ptc1) triggers Smo accumulation within the ciliary membrane and activation of Shh signaling. Smo is transported laterally from the PM into the ciliary membrane through the diffusion barrier at the base of the cilium (Rohatgi et al., 2007; Milenkovic et al., 2009). After ciliary membrane proteins reach the PM, some undergo endocytosis followed by polarized exocytosis at the periciliary membrane. For example, the ciliary localization of the membrane protein Kim1 involves Rab5-dependent endocytosis from the PM via the recycling pathway, followed by Rab8-dependent polarized exocytosis to the base of the cilium (Boehlke et al., 2010). In many cell types or organisms, such as *Trypanosoma* and mammalian fibroblasts, the proximal region of the cilium is surrounded by a cytoplasmic invagination of the periciliary membrane, known as the ciliary pocket (CiPo). The CiPo is enriched in clathrin-coated pits, which make it a hotspot for exocytosis and endocytosis of vesicles for the delivery and retrieval of ciliary membrane component (Pedersen et al., 2016). This “recycling pathway” is likely be regulated by distinct sets of proteins. The endosome maturation factors Rabenosyn-5 (RABS-5)/VPS45 and Caveolin-1 regulate the process of periciliary-derived endocytic vesicle and ciliary membrane homeostasis (Scheidel et al., 2018). Transforming growth factor beta (TGF-β) signaling is regulated by clathrin-dependent endocytosis for the control of receptor localization and activation of SMAD2/3 and ERK1/2 at the ciliary base (Clement et al., 2013). BLOC1 is also required for selective membrane protein trafficking from endosomes to primary cilia (Monis et al., 2017). Moreover, PI3K class II alpha (PI3K-Cα) is enriched in the pericentriolar endocytic compartment (PRE) at the base of primary cilia, where it regulates the formation of a PtdIns3P pool at the PRE required for Rab11 and Shh pathway activation (Franco et al., 2014).

The second destination for vesicles carrying ciliary membrane proteins is the periciliary membrane, a specialized membrane region distinct from the PM and ciliary membrane. Some ciliary membrane proteins are transported directly from vesicles to the periciliary membrane. In polarized epithelial cells, the periciliary membrane is characterized by enrichment of galectin-3, exclusion of cortical actin cytoskeleton, and high endocytosis and exocytosis (Vieira et al., 2006; Molla-Herman et al., 2010; Francis et al., 2011; Stoops et al., 2015). The subsequent fusion of vesicles to the periciliary membrane is mediated by SNAREs. The vesicular membrane contains v-SNAREs, such as Vamp3 and Vamp7 (Szalinski et al., 2014; Finetti et al., 2015), whereas SNAP25, SNAP29, and Syntaxin3 localize to the periciliary membrane; these proteins form the t-SNARE complex (Baker et al., 2008; Mazelova et al., 2009b; Lu et al., 2015).

## The Entrance of Ciliary Membrane Proteins Into Cilia Through Diffusion Barrier

The diffusion barrier is the membrane portion of the ciliary TZ, which is the zone in which BBS (related to Bardet-Biedl syndrome), MKS (related to Meckel-Gruber syndrome), JS (related to Joubert syndrome), and NPHP (related to Nephronophthisis) are located (Cevik et al., 2013; Jensen et al., 2015; Lambacher et al., 2016). In 2016, Dennis et al. isolated the TZ from *Chlamydomonas* for the first time and identified its protein composition, which includes the classical TZ proteins CEP290, NPHP1, NPHP4, MKS1, MKS3/TMEM67, MKS6, Tectonic, AHI1, and BBS. TZ also contains a series of ESCRT-complex proteins, including VPS4, VPS23, VPS28, VPS37, VPS60, and ALIX. In addition, TZ includes two subunits of katanin, POC2 and MOT51, and the cyclins CDKA1 and CYCA1 (Diener et al., 2015). Subsequently, 192 candidate TZ proteins were identified by immunoprecipitation enrichment in *Trypanosoma*, including the MKS complex and various conserved TZP proteins (Dean et al., 2016). These results suggest that the proteins located in the TZ are highly conserved between species, but their roles in the formation of diffusion barriers, and how they coordinate with each other to regulate the function of diffusion barriers, remain unclear.

A crucial function of the diffusion barrier is distinguishing ciliary membrane from PM and maintaining the specific compositions of proteins and lipids in ciliary membrane. Studies in mammalian cells indicate that membrane proteins can shuttle between PM and ciliary membrane. This process involves three main complexes (Figure 1): the IFT-A, IFT-B, and BBSome complexes (Nachury, 2018). The IFT-A complex regulates the transport of ciliary membrane proteins into cilia, whereas the BBSome complex mediates the transport of ciliary membrane proteins out of cilia. When a cilium-related signal is activated, as in Hedgehog signaling, the responding ciliary membrane receptors bind to the IFT-A complex and are transported into the cilia with the help of the membrane protein TULP3. The IFT-A complex subunits IFT121 and IFT144 are essential to the accumulation of GPCRs in cilia. In the meantime, the responding membrane proteins that need to be transported out of cilia interact with the BBSome complex, and are then exported through the action of the IFT-B complex, in a process mediated by the membrane proteins ARL6 and β-arrestin2. When a subunit of the BBSome complex is mutated, GPCRs cannot be smoothly transported back to the cell body and are secreted to the outside of the cell along with the ciliary ectosomes. This is another way in which ciliary membrane proteins can exit cilia (Nachury, 2018).

Studies in *Caenorhabditis elegans* indicate that the TMEM-107/231, MKS, and NPHP complexes are involved in regulating the function of the diffusion barrier (Cevik et al., 2013; Jensen et al., 2015; Lambacher et al., 2016). For instance, MKS-5 (whose mammalian homolog is Rpgrip 1L/Rpgrip 1) is necessary to the formation of the diffusion barrier in three respects: (1) it facilitates the accurate localization of other known TZ proteins; (2) it is required for the formation of the TZ ultrastructure, including the Y-linker structure between the axoneme and diffusion barrier; (3) diffusion barrier-localized MKS-5 and other proteins exclude PIP2 from entering into the cilia, ensuring that the ciliary membrane maintains its specific lipid composition (Jensen et al., 2015). The function of the diffusion barrier is regulated by other specific proteins. For example, the GTPase Septin 2, a component of the diffusion barrier localized to the junction between ciliary membrane and PM in both mammalian cells and *Chlamydomonas*, helps regulate the transport of ciliary membrane receptors. When Septin 2 expression is knocked down, most cells have no cilia, a few have short cilia, and the accumulation of the ciliary membrane protein Smo in cilia is reduced; subsequently, the transduction of the Sonic hedgehog signal is disturbed (Hu et al., 2010). When the mouse Septin 4 gene is mutated, the diffusion barrier of sperm tail does not form and the localization of flagellar membrane proteins is altered (Kwitny et al., 2010). NPHP4 is stably incorporated into the distal part of the ciliary TZ. The abundances of certain membrane proteins are greatly reduced in *nphp4* mutant cilia, and cellular housekeeping proteins larger than 50 kDa are not excluded from these cilia. Thus, NPHP4 functions as an essential part of a barrier that regulates both the membrane and soluble protein compositions of cilia (Awata et al., 2014). The Kharon protein of *Trypanosoma* localizes at the ciliary base and interacts with calcium channels to regulate their transport to cilia (Sanchez et al., 2016). Two Rab-effector-related proteins, Rab-interacting lysosomal protein-like 1 (Rilpl1) and Rilpl2, regulate protein localization in ciliary membrane. Depletion of Rilpl1 and Rilpl2 results in the accumulation of signaling proteins (such as fibrocystin) in the ciliary membrane and prevents proper epithelial cell organization in three-dimensional culture (Schaub and Stearns, 2013).

In summary, when the function of the diffusion barrier is abnormal, several characteristics are observed at the cellular level: (1) cilium growth is inhibited; (2) the ability of cells to sense external signals is reduced or lost; (3) the transport of membrane proteins is abnormal; and (4) the lipid composition of the ciliary membrane changes (Hunnicutt et al., 1990; Jensen et al., 2015). These characteristics lay the foundation for screening mutants that are defective in diffusion barrier formation and regulation through forward-genetic methods.

## The Regulation of Ciliary Membrane Homeostasis by Ciliary Ectosome Secretion

Apart from transport into or out of cilia, the homeostasis of ciliary membrane proteins is also regulated by the secretion of ciliary vesicles (Figure 1). These small (50–200 nm) vesicles bud from the ciliary membrane and are secreted to the extracellular environment (Wood et al., 2013; Wang et al., 2014). The size and manner of secretion of these vesicles are similar to those of ectosomes secreted from the PM, and they are therefore known as ciliary/flagellar ectosomes (Wood et al., 2013). The secretion function of cilia was first discovered in the model organism *Chlamydomonas*, in which the ciliary ectosomes carry bioactive enzymes that digest the mother cell wall and release the daughter cells during reproduction (Wood et al., 2013).

During mating, both ciliary touching and signaling depend on a protein called SAG1. When the sex cells’ cilia touch, SAG1 protein abundance increases rapidly in the cilia. Afterward, SAG1 disappear from cilia without returning to the PM; instead, it is packaged within ciliary ectosomes and then shed from cilia into the external environment. Isolated ciliary ectosomes are capable of activating cilium-generated signaling (Cao et al., 2015). Cilia of *Chlamydomonas* vegetative cells and gametes both undergo constitutive membrane shedding of ciliary ectosomes. The work of Denler (Dentler, 2013) shows that vegetative cells shed a minimum of 16% of their flagellar membrane per hour, equivalent to a complete flagellar membrane being released every 6 h or less. Work by Cao et al. (2015) shows that nearly the entire cellular complement of pre-existing SAG-C65-HA is shed in around 3 h in gametes. Thus, receptor-signaling-induced shedding of ciliary ectosomes is much more rapid in vegetative cells than in normal growing cells. In future studies, it will be interesting to explore the distinct regulatory mechanism controlling these effects.

ESCRT proteins mediate ectosome release and thereby influence flagellar shortening and mating (Long et al., 2016). Although the ciliary ectosomes are derived from ciliary membrane, their protein composition is different. Ciliary ectosomes are enriched in a subset of ciliary membrane proteins, proteases, proteins from the endosomal sorting complex required for transport, small GTPases, and ubiquitinated proteins (Long et al., 2016). These results suggest that the secretion of ciliary ectosomes regulates the homeostasis of ciliary membrane proteins and is in turn strictly regulated.

The turnover of ciliary membrane proteins through ciliary ectosome secretion is conserved in many species. In *C. elegans*, the ciliated sensory neurons shed and release extracellular vesicles (ECVs) (Wang et al., 2014). The biogenesis of ECVs occurs via budding from the periciliary membrane and not via fusion of multivesicular bodies. ECVs isolated from the cultured plate of wild-type worms function in cellular communication and induced mating-related male tail-chasing behavior (Wang et al., 2014). Bloodstream African *Trypanosomes* produce membranous nanotubes that originate from the flagellar membrane and disassociate into free ECVs. *Trypanosome* ECVs contain several flagellar membrane proteins, mediate non-hereditary virulence factor transfer, and cause host erythrocyte remodeling that induces anemia (Szempruch et al., 2016).

In mammalian cells, when activated GPCRs fail to undergo BBSome-mediated retrieval from cilia back into the cell, these GPCRs concentrate into ciliary ectosomes and are released from the cilia. Actin and the actin regulators Drebrin and Myosin 6 mediate this process. Therefore, signal-dependent ciliary ectosome release is a selective and effective process that removes activated signaling receptors from cilia (Nager et al., 2017). Moreover, primary cilia vesicle release is promoted by INPP5E loss and growth stimulation. INPP5E loss in cilia triggers actin polymerization in primary cilia, which excises cilia tips in a process known as decapitation. Decapitation induces mitogenic signaling and constitutes a molecular link between the cilium life cycle and the cell-division cycle (Phua et al., 2017).

In future studies, it will be useful to expand the investigation of ciliary secretion function. These studies can further elucidate the molecular mechanism and regulation of ciliary ectosome release, the cellular processes and corresponding mechanisms involved in ciliary ectosome secretion, and identify more of the genes involved in regulating the ciliary secretion function.

## Conclusion

The sensory function of cilia is mainly dependent on ciliary membrane proteins. In addition to investigating the processes controlling the activity of these proteins *in situ*, it is important to assess how cells regulate the amounts of membrane proteins localized in cilia. Thus, it is critical to identify factors involved in the trafficking of ciliary membrane proteins to their destination. Based on current research into the processes that build and maintain the homeostasis of ciliary membrane proteins in cilia, several areas are worthy of further investigation: (1) the coordination of different organelles, such as the PM, ER, and Golgi, during ciliary membrane protein transport; (2) the roles of lipids in sorting, localization, and transport of ciliary membrane proteins; (3) the regulation and complete composition of the diffusion barrier; (4) the molecular mechanism of IFT involved in moving the ciliary membrane proteins in cilia; and (5) the molecular mechanism of ciliary ectosome secretion in regulating the turnover of ciliary membrane proteins. Studies in these areas will deepen our understanding of the pathology of ciliopathies resulting from defects in ciliary membrane traffic.

## Author Contributions

HL and KH conceived and wrote the review together.

## Conflict of Interest

The authors declare that the research was conducted in the absence of any commercial or financial relationships that could be construed as a potential conflict of interest.
